# Structural phases and electrochemical properties of 2D MoS_2_ for supercapacitor applications

**DOI:** 10.1039/d5ra08612d

**Published:** 2026-01-22

**Authors:** Seyoum Abebayehu Getaneh, Arnaud Magrez, Getachew Adam Workneh

**Affiliations:** a Department of Industrial Chemistry, Addis Ababa Science and Technology University P.O. Box 16417 Addis Ababa Ethiopia getachew.adam@aastu.edu.et; b Sustainable Energy Center of Excellence, Addis Ababa Science and Technology University P.O. Box 16417 Addis Ababa Ethiopia seyoumab5@gmail.com; c Crystal Growth Facility, Institute de Physique, Ecole Polytechnique Fédérale de Lausanne Lausanne Switzerland

## Abstract

Supercapacitors (SCs) are becoming popular as electrochemical energy storage devices because they have a high-power density, fast charge and discharge rates, and good cycling stability. The materials used in the electrodes have a big effect on how well supercapacitors work overall. Two-dimensional molybdenum disulfide (2D MoS_2_) has gotten a lot of attention among a number of other electrode materials, such as carbon-based materials, metal oxides, and conducting polymers. This is mostly because of its layered structure, large surface area, and ability to change its electrical properties. MoS_2_ shows strong links between structure and properties. The bulk form of MoS_2_ has an indirect bandgap of about 1.2 eV, while the monolayer form has a direct bandgap of about 1.8 eV. This difference in bandgap affects how well it works in optoelectronic and electrochemical applications. MoS_2_ also comes in different forms, such as the semiconducting 2H, the metallic 1T, and the rarer 3R. Each of these has its own unique atomic structure. Alkali metal intercalation, mechanical strain, or doping with elements like Re, Tc, or Mn can cause phase changes from 2H to 1T. This makes the material more conductive and improves the performance of supercapacitors. This review thoroughly examines recent advancements in phase engineering and the electrochemical performance of 2D MoS_2_-based supercapacitor electrodes, highlighting the effects of intrinsic modifications (phase engineering) and extrinsic modifications (composite formation) on the charge-storage characteristics of MoS_2_.

## Abstract 2

2D MoS_2_ exists mainly in semiconducting 2H and metallic 1T phases and is widely studied among TMDs. Phase engineering from 2H to 1T improves conductivity, charge transport, and supercapacitor performance.

## Graphical abstract

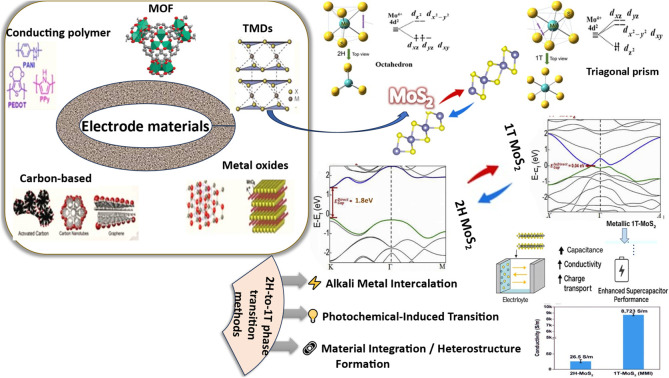

## 1 Introduction

The rapid rise in global warming and environmental pollution caused by the use of fossil fuels, driven by population growth and the growing demand for portable electronics and electric vehicles, has heightened the importance of finding clean, cheap, portable, and efficient alternative energy sources. Solar and wind energy are environmentally friendly; however, they are not always available due to weather conditions and the time of day.^1,2^ As a result, there is a pressing demand for advanced energy storage systems capable of capturing and stabilising the output from these variable sources. Electrochemical energy storage (EES) technologies are at the forefront of addressing this challenge, providing efficient means to store and release energy on demand. Among the various EES systems, batteries, supercapacitors, and fuel cells are the most prominent.^3,4^ These systems play critical roles in consumer electronics, electric vehicles, and grid-scale storage.

Batteries, such as lithium-ion and nickel-cadmium, are valued for their high energy density (10–1000 Wh kg^−1^), making them suitable for long-term energy storage. However, their performance is hindered by slow ion and electron transport, leading to resistive heating and, in some cases, dendrite formation during high-power operation. These issues compromise both efficiency and safety, as evidenced by documented failures in electric vehicles and aircraft.^3^

In contrast, supercapacitors (SCs) have gained popularity due to their high power density (>10 kW kg^−1^), quick charge–discharge capability, and great cycling stability (reaching 100 000 cycles).^5^ SCs store energy *via* electrostatic interactions or rapid surface redox processes, allowing them to outperform batteries in situations demanding high power delivery. Supercapacitors improve their performance by adopting electrode materials with a high specific surface area (SSA), which increases charge storage and reduces diffusion distances.^6,7^ Conventional capacitors possess capacitance in the micro-to milli-Farad range; however, supercapacitors can attain thousands of Farads per unit due to their sophisticated materials and design. In contrast to batteries, which store charge *via* bulk ion intercalation, supercapacitors accumulate charge at or near the surface of electrode materials, yielding enhanced power density for equivalent device volume.^8^

**Fig. 1:**
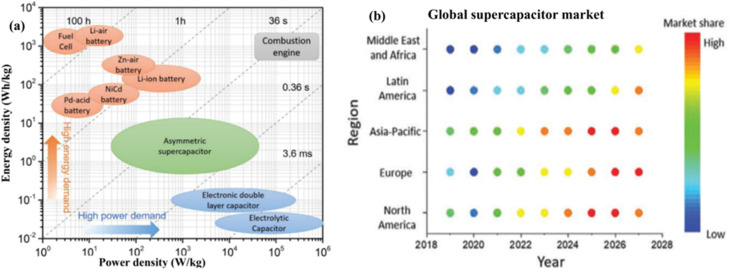
(a) Ragone plot demonstrating the relationship between specific power and specific energy across various electrical energy-storage technologies. Adapted from ref. 3 with permission from the American Chemical Society, *Chemical Reviews*, copyright © 2018, and (b) global supercapacitor market, reproduced from ref. 16 with permission from the Royal Society of *Chemistry, Energy & Environmental Science*, copyright © 2021.

Fuel cells, batteries, and supercapacitors are all forms of energy storage, but their respective power densities and energy capacities limit their potential uses. Because of their low power output and high energy density, fuel cells are preferable for stationary and hybrid applications where steady-state energy delivery is required, as shown in the Ragone plot (Fig. 1).^9^ Batteries offer a balance, with high energy densities but limited power performance and slow charging rates. Despite their poor power density, safety concerns, and limited cycle life, lithium-ion batteries (LIBs) have dominated the market since their commercial introduction in 1990.^10,11^ Supercapacitors have arisen as an alternative to LIBs in order to circumvent these drawbacks. Even though their use is limited in some applications because of their low energy density, they have a long lifespan and fast response. Investigating ways to close this energy gap is of the utmost importance. To increase energy density while keeping power output high, one potential approach is the creation of asymmetric supercapacitors (ASCs), which merge capacitor-type electrodes with battery-type faradaic materials.^12,13^ Unlike electric double-layer capacitors (EDLCs), which store charge electrostatically, ASCs employ redox-active materials on one electrode and capacitive materials on the other. The operating voltage window is expanded, and energy storage capacity is increased using this hybrid technique. Metal oxide-carbon, conducting polymer-carbon, and transition metal dichalcogenide (TMD)-carbon hybrids are some of the combinations that can be used to create ASCs.^14,15^

**Fig. 2:**
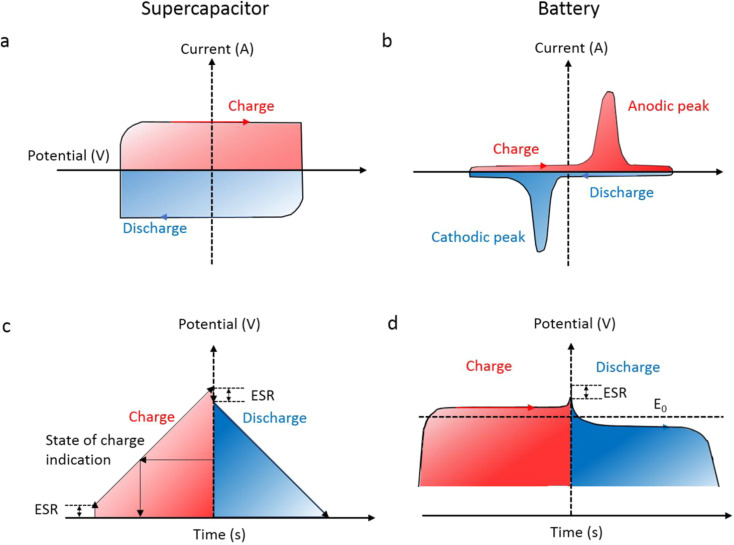
Analysis of the electrochemical characteristics of a standard supercapacitor *versus* a battery: (a) and (c) represent CV curves, while (b) and (d) illustrate GCD curves, adapted from ref. 3 with permission from the American Chemical Society, *Chemical Reviews*, copyright © 2018.

During constant current charging and discharging, SCs show a linear voltage–time relationship, which reflects a direct correlation between stored charge and voltage. Fig. 2c shows the galvanostatic charge–discharge (GCD) curve of an SC, which differs from the voltage plateaus observed in batteries (Fig. 2d). It often has rectangular cyclic voltammetry (CV) profiles (Fig. 2a), whereas batteries show clear peaks (Fig. 2b) as a result of faradaic reactions.^17,18^ These differences in charge storage mechanisms lead to different units of measurement, capacitance (F) for SCs and capacity (mAh) for batteries. In GCD analysis, Equivalent Series Resistance (ESR) manifests as a sudden voltage drop at the onset of the discharge curve. For supercapacitors (Fig. 2c), this drop is more pronounced due to their inherently higher ESR, arising from electrode/electrolyte interfaces and porous structures. In batteries (Fig. 2d), the ESR is typically lower, but still contributes to efficiency loss and heat generation. Comparing both, lower ESR is critical for high-power performance, and its magnitude directly affects the charge/discharge efficiency and energy delivery rate.

## 2 Historical development and classification of supercapacitors

### 2.1 Historical development of supercapacitors

The development of supercapacitors dates back to the mid-18th century, notably with the invention of the Leyden jar by Ewald Georg von Kleist and Pieter van Musschenbroek between 1745 and 1746, marking the first demonstration of a capacitor.^19,20^ This apparatus laid the foundation for understanding charge storage at the solid–liquid interface, a concept that predates the invention of batteries by over a century. In 1853, Hermann von Helmholtz presented the first model of the electric double-layer (EDL), clarifying the processes of charge storage at the interfaces between electrodes and electrolytes.^21^ Subsequently, Gouy (1910) and Chapman (1913) developed a diffuse layer model, which Stern (1924) subsequently modified into the Gouy–Chapman–Stern model, effectively integrating the principles of both Helmholtz and the diffuse layer.^22,23^

The modern history of supercapacitors can be traced back to 1954, when H. I. Becker of General Electric patented an electrochemical capacitor built with porous carbon electrodes, an idea that, despite its novelty, never progressed to commercial use. A notable step forward occurred in 1966, when Robert Rightmire at SOHIO developed a capacitor based on a non-aqueous electrolyte, allowing the device to operate at considerably higher voltages of about 3.4–4.0 V.^24^ The field advanced further in 1971 with the identification of pseudocapacitance in RuO_2_ electrodes, which introduced faradaic charge transfer as a mechanism for enhanced energy storage.^25^ By 1978, the Nippon Electric Company (NEC) released the first device marketed as a “Super-Capacitor,” primarily intended for backup power applications.^26^

**Fig. 3:**
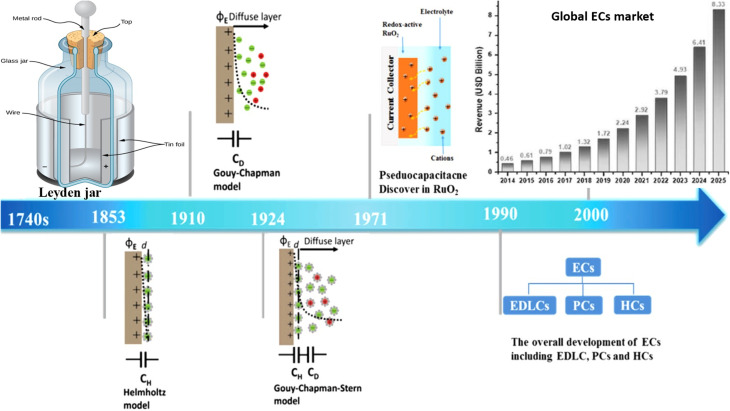
Chronological account of the evolution of electrochemical capacitors, adapted from ref. 3 with permission from the American Chemical Society, *Chemical Reviews*, copyright © 2018.

Currently, supercapacitors encompass electrical double-layer capacitors (EDLCs), pseudocapacitors (PCs), and hybrid capacitors (HCs). Contemporary supercapacitors are capable of reaching capacities in thousands of farads and can manage charge–discharge currents that vary from tenths to hundreds of amperes (Fig. 3).^27–29^

### 2.2 Classification of supercapacitors according to their charge storage mechanisms

**Fig. 4:**
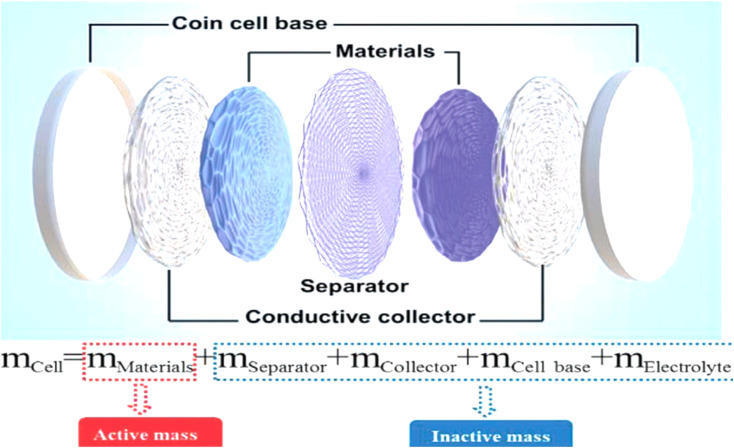
Schematic of an EDLC coin cell, showing electrode materials, separator, conductive collector, and electrolyte, reproduced from ref. 31*Advanced Science*, 2021.

A comprehensive supercapacitor assembly has two electrodes, an electrolyte, and a separator component, as seen in Fig. 4.^30,31^ The separator enables ion permeability while effectively preventing direct electrical contact between the electrodes.^32,33^ As stated earlier, supercapacitors are classified into three main types based on their methods of energy storage: EDLCs, PCs, and HCs.^34,35^

#### 2.2.1 Electric double‐layer capacitors

EDLCs store charge electrostatically *via* a non-faradaic mechanism. They typically consist of two porous electrodes made of activated carbon, separated by an electrolyte and a physical separator to maintain electrical isolation. The performance of EDLCs is dependent on the surface area of the porous electrodes, as greater surface area allows more charge to be stored per unit voltage. As electrode pore size decreases to near the electrolyte ion size, the capacitance and specific energy typically increase, due to enhanced ion accessibility and greater surface area.

**Fig. 5:**
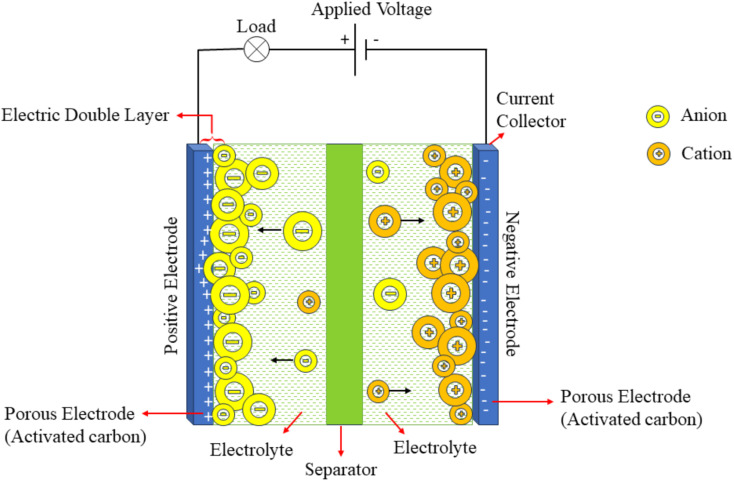
Schematic diagram of EDLCs, reproduced from ref. 36 (*World Electr. Veh. J.*, 2024), licensed under CC BY.

Upon application of voltage to the electrode terminals in EDLCs, charge accumulation occurs on the surfaces of the electrodes (Fig. 5). The resulting potential difference causes attraction between oppositely charged electrodes. Electrolyte ions move through the separator into the pores of the electrode carrying the opposite charge and form an electric double layer on the surface of electrode, where energy is stored through electrostatic separation of positive and negative charges rather than by chemical reactions or ion recombination. This double layer is established as oppositely charged ions assemble at the surface of each electrode.

A key feature of EDLCs is the absence of charge transfer reactions at the interface, resulting in the absence of faradaic processes. This facilitates rapid charge and discharge cycles along with exceptional stability. EDLCs' specific capacitance is primarily governed by the electrode material's accessible surface area and the surface properties of the carbon-based materials.^37^Eqn (1) can be employed to estimate the capacitance of an EDLC electrode.^38,39^ A denotes the effective surface area of the electrode that is available for interaction with electrolyte ions.1
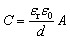


#### 2.2.2 Pseudocapacitors

**Fig. 6:**
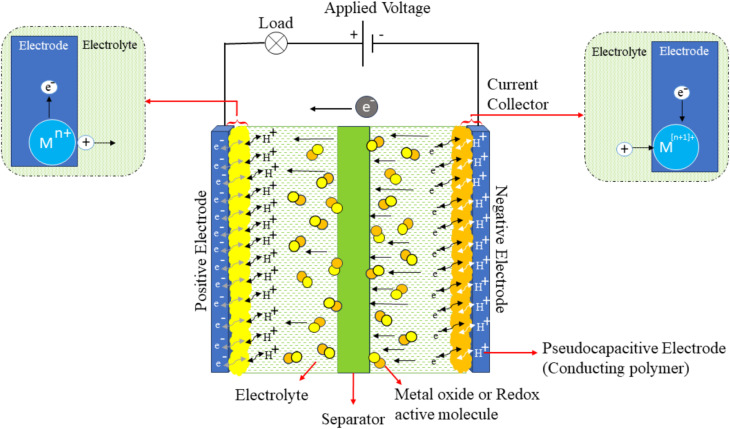
Schematic illustration of a PC device, showing the charge storage mechanism involving faradaic redox reactions at the electrode–electrolyte interface, reproduced from ref. 36 (*World Electr. Veh. J.*, 2024), licensed under CC BY.

Unlike EDLCs, the electrode materials in PCs store charges through fast and reversible oxidation/reduction (faradaic), characterised by swift and reversible redox reactions on the surfaces of the active materials.^40,41^ This behaviour is attributed to a change in the oxidation state of the electrode material, driven by electron exchange. RuO_2_ stands out as the pioneering electrode material documented to display pseudocapacitive characteristics.^25,42^ The energy storage mechanism *via* pseudocapacitance exhibits a characteristic electrochemical behaviour that lies between the purely electrostatic characteristics of EDLCs and a bulk battery-type material. In pseudocapacitors, proton (H^+^) ions are incorporated into the charge storage mechanism, especially in aqueous electrolytes like sulphuric acid. The H^+^ ions are intrinsically present due to acid dissociation and move throughout the electrolyte during operation. Upon the application of voltage, electrons traverse the external circuit, while protons migrate through the electrolyte, engaging in rapid and reversible redox processes at the electrode–electrolyte interface. This faradaic technique facilitates elevated pseudocapacitance, especially in electrodes composed of conducting polymers (CPs) or transition metal oxides (TMOs) like MnO_2_. The schematic diagram shown in Fig. 6 generally contains two pseudocapacitive electrodes, a separator, and current collectors, enabling both ion transport and electron conduction.

These processes drive charge movement across the electric double layer, producing a faradaic current within the supercapacitor cell. PCs are prominent in their elevated electrochemical pseudocapacitance, which is dependent on the applied voltage. Typically, they consist of materials like metal oxide, with high capacitance or conductive polymers. The faradaic mechanisms improve electrochemical performance by boosting both specific capacitance and energy density. Despite their advantage in energy density, pseudocapacitors generally exhibit lower power density and shorter cycle life.^36^

#### 2.2.3 Hybrid capacitors

**Fig. 7:**
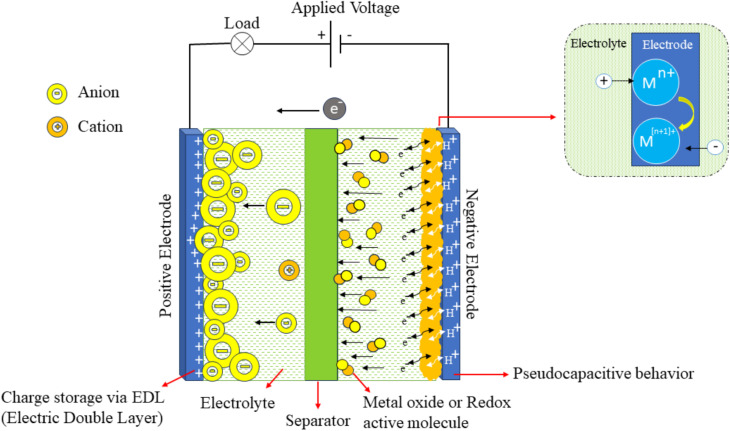
Schematic representation of a hybrid supercapacitor, composed of one EDLC-type carbon-based electrode and one pseudocapacitive electrode, reproduced from ref. 36 (*World Electr. Veh. J.*, 2024), licensed under CC BY.

Recent research has focused on designing and developing hybrid capacitors to tackle the problem of supercapacitors having a lower energy density than batteries and fuel cells. Hybrid capacitor technologies are a strategic new idea in the field of electrochemical energy storage. They aim to combine the best features of EDLCs and PCs.^17,43^ It generally consists of one electrode composed of EDLC-type carbon-based materials and another electrode made from a pseudocapacitive material, which may include TMOs or CP.^1,2^ EDLCs have a poor energy density, but their non-faradaic charge storage method gives them a high power density and a long cycle life.^44^ Pseudocapacitors, which use faradaic redox processes, give more energy storage capacity but may sacrifice cyclic stability and rate performance.^40^Fig. 7 shows how HCs store charge. This technique tries to improve the overall energy density of the device by combining materials with high specific capacitance and allowing it to work at higher cell voltages.

In hybrid capacitors, particularly asymmetric configurations, the two electrodes typically rely on different charge-storage mechanisms. The negative electrode usually contains a carbon-based EDLC material, whereas the positive electrode contains a pseudocapacitive component such as a transition-metal oxide (TMO) or a conducting polymer (CP). Each material operates within its own stable potential range in the selected electrolyte. By selecting electrodes whose potential windows complement one another, the overall cell voltage can be significantly extended. This difference in potential between the two electrodes is what ultimately broadens the operating voltage window, a parameter directly linked to achieving higher energy density, as shown in eqn (2).^45^ Energy density is a key factor in designing how well supercapacitors work electrochemically. Increasing the operational voltage range is a straightforward and effective way to increase the energy density (*E*) of a supercapacitor device. Consequently, designing electrode materials that can maintain wide and stable potential windows has become a practical and effective route to improving device-level energy storage.2
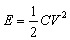
where *C* represents the capacitance, and *V* denotes the operating voltage window for the cell. According to this expression, a doubling of voltage leads to a quadrupling of energy density while maintaining the same capacitance value. Hence, a carefully engineered hybrid capacitor can deliver superior energy density, making it suitable for applications that require both efficient energy storage and rapid power delivery. Moreover, hybrid systems that integrate both EDLC and faradaic charge-storage behaviors open new opportunities for developing next-generation, high-performance energy storage technologies (Table 1).

**Table 1:** Summary of EDLCs, PCs, and HCs

Feature	EDLCs	PCs	HCs
Voltage & power operation	High voltage and high-power operation	Low-voltage operation is limited by electrochemical processes and the voltage at which solvent decomposition occurs	Increased cell voltage
Electrode materials	Carbon-based materials	MOs and CPs	A combination of carbon with MOs or CPs
How is charge stored?	Electrochemical double layer; non-faradaic process	Faradaic redox reactions	Both faradaic and non-faradaic processes

### 2.3 Electrochemical characterization parameters of supercapacitors

Electrochemical characterization plays a central role in assessing supercapacitors, as it provides insight into their charge-storage mechanisms, overall performance, and operational efficiency. These analyses also inform the rational design and optimization of electrode materials. Typically, supercapacitor performance is evaluated using cyclic voltammetry (CV), galvanostatic charge–discharge (GCD), and electrochemical impedance spectroscopy (EIS), techniques that directly track the relationships among current, voltage, and time.^46,47^ From these fundamental measurements, a range of critical parameters, including specific capacitance, energy and power densities, coulombic efficiency, equivalent series resistance (ESR), relaxation time constant, and long-term cycling stability, can be determined.^48^

**Fig. 8:**
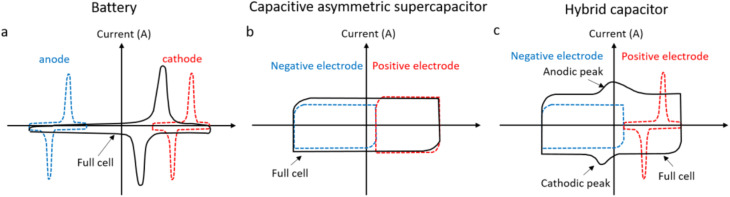
Cyclic voltammograms visually distinguish the storage mechanisms, characteristics of (a) a battery, (b) a capacitive asymmetric supercapacitor, and (c) a hybrid capacitor, adapted from ref. 3 with permission from the American Chemical Society, *Chemical Reviews*, copyright © 2018.

CV provides insight into the charge-storage mechanism by sweeping the electrode potential linearly with time and measuring the corresponding anodic and cathodic currents. The shape of the voltammogram differentiates between double-layer capacitive behaviour (rectangular CV curves) and pseudocapacitive processes (redox peaks or broadly distributed peaks).^49^ EDLC-type MoS_2_ typically exhibits near-rectangular curves, whereas 1T-MoS_2_ and defect-rich or composite MoS_2_ structures show quasi-rectangular shapes with redox humps due to surface redox reactions, as shown in Fig. 8. Quantitatively, the specific capacitance from CV is obtained by integrating the enclosed area under the CV curve:^48^3
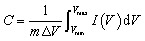
where *I*(*V*) is the instantaneous current at a given potential *V*, and *V*_min_ and *V*_max_ define the lower and upper limits of the potential window, respectively.

CV also allows for estimation of the operating voltage window, reversibility, and kinetics, while scan-rate-dependent measurements help separate capacitive (surface-controlled) and diffusion-controlled contributions. Schematic diagrams of EDLC-type, pseudocapacitive, and battery-type CV curves have been included in Fig. 8 to visually distinguish the storage mechanisms.

**Fig. 9:**
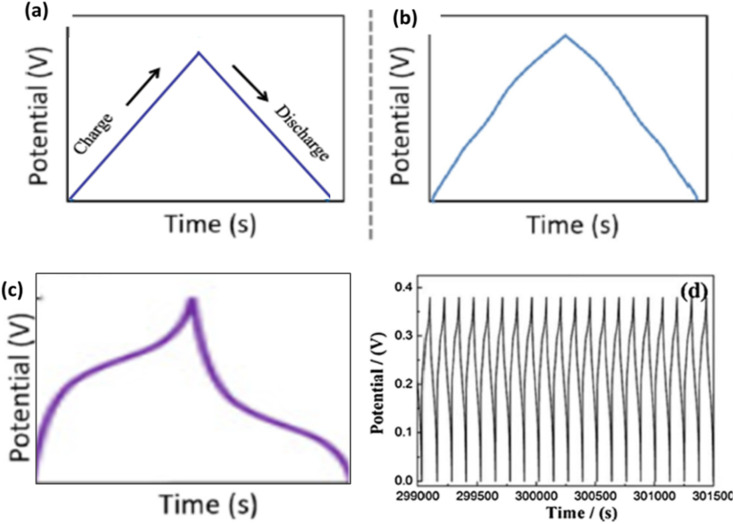
An illustration of GCD curves of (a) EDLCs with linear potential change, (b) pseudocapacitors exhibit a non-linear curve, (c) battery type, and (d) GCD curves for stability test, adapted from ref. 51 (*Energy & Environmental Materials*, 2019) with permission from John Wiley & Sons, copyright © 2019.

Galvanostatic charge–discharge (GCD) is the most widely used technique for evaluating specific capacitance, energy efficiency, and cycling behaviour of SC electrodes.^50^ A SC is charged and discharged at a fixed current, and the resulting potential–time profile provides direct information on capacitance, IR drop, reversibility, and long-term stability. In EDLCs, the potential–time profile is linear, while in pseudocapacitive or battery-type systems, the curves exhibit nonlinear regions or plateaus associated with faradaic processes, as shown in Fig. 9.

The degree of curvature, the magnitude of the IR drop at the beginning of discharge, and the symmetry between charge and discharge slopes all provide valuable insights into kinetic limitations and electrical resistances present in the system. From GCD curves, the specific capacitance (*C*, F g^−1^) can be calculated using:4
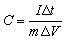
where *I* is the applied current (A), Δ*t* is the discharge time (s), *m* is the mass of active electrode material (g), and Δ*V* is the potential window excluding the instantaneous IR drop. This parameter reflects how efficiently the electrode stores charge per unit mass.

Energy density (*E*, Wh kg^−1^) and power density (*P*, W kg^−1^), key practical metrics for real-world supercapacitor applications, are obtained from the following expressions:5
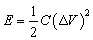
6
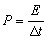


Energy density shows how much energy the material can store, while power density describes how rapidly the stored energy can be delivered. Coulombic efficiency (*η*) is another performance indicator obtained directly from GCD curves, measuring how effectively a device can recover the charge it has stored:7
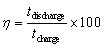


High coulombic efficiency (close to 100%) reflects excellent reversibility, low polarization, and minimal parasitic reactions. GCD testing is also the standard method for evaluating long-term cycling stability. By repeatedly charging and discharging the electrode for hundreds to thousands of cycles, the retention of capacitance over time can be determined (Fig. 9d). A retention above 80–90% after several thousand cycles indicates a structurally robust, electrochemically stable electrode material.8
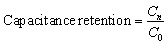
where *C*_0_ and *C*_*n*_ are the initial and *n*th cycle capacitances.

**Fig. 10:**
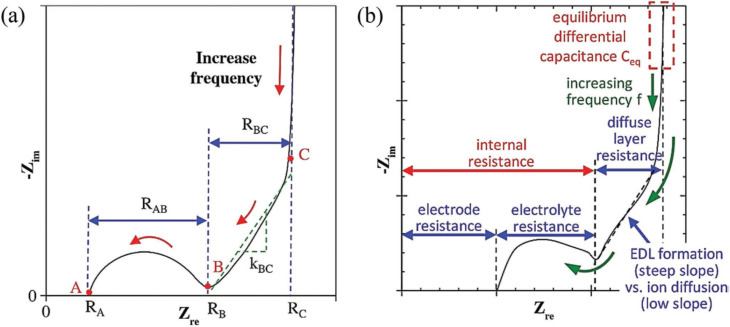
Schematic of a typical Nyquist plot for (a) EDLC supercapacitors and (b) different types of resistances in the cell, reproduced from ref. 53 (*Advanced Energy Materials*, 2020) with permission from John Wiley & Sons, copyright © 2020.

Electrochemical impedance spectroscopy (EIS) provides frequency-dependent insights into charge-transfer resistance, ion diffusion, and interfacial behavior. Using a small alternative current (AC) perturbation (typically 5 mV), impedance is measured across a wide frequency range to generate Nyquist and Bode plots.^52^ The intercept at the real axis gives the solution resistance (*R*_s_), while the semicircular diameter corresponds to charge-transfer resistance (*R*_ct_). EIS tests in a three-electrode system frequently measure solution resistance (*R*_s_), charge transfer resistance (*R*_ct_), and Warburg impedance (*W*), which are represented by Nyquist plots in Fig. 10. Warburg impedance (*W*) is another component of impedance that results from the diffusion of molecules or redox species. Warburg impedance varies in frequency, with low values at high frequencies where reactants move short distances and greater values at low frequencies where reactants diffuse further. It is shown by a 45° sloped line on Nyquist plots and a 45° phase shift on Bode charts. Capacitance can be extracted from the imaginary impedance using:9
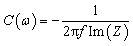


evaluated typically at the lowest frequency or at the 45° phase angle. Analysis of EIS parameters enables identification of rate-limiting processes, optimization of electrode architectures, and comparison of intrinsic conductivity across different MoS_2_ phases and composites.

## 3 Electrode materials in supercapacitors

Recently, significant efforts have been made to explore and enhance materials suitable for electrodes. These materials are anticipated to provide enhanced electrical conductivity, thermal resistance, and robust chemical stability. Furthermore, they must demonstrate a substantial specific surface area (SSA), facilitate efficient faradaic charge transfer, provide corrosion resistance, be ecologically friendly, and maintain cost-effectiveness.^54^ Furthermore, the capacity of electrode materials to promote faradaic charge transfer is essential for improving capacitance performance. The accessibility of electrolytes, which greatly affects specific capacitance, is primarily determined by the morphology of the electrode, the distribution of pore sizes, and the geometry of the pores. The structural characteristics influence the efficiency with which electrolyte ions can infiltrate the electrode material and reach active areas for charge storage.^55^

### 3.1 Commonly used electrode materials in supercapacitors

Supercapacitors utilise several electrode materials, each engineered to enhance performance based on the specific application. The comparative analysis of principal electrode material families, encompassing carbon materials, conducting polymers, transition-metal oxides/hydroxides, layered double hydroxides (LDHs), MXenes, black phosphorus (BP), and two-dimensional transition metal dichalcogenides (TMDs) like MoS_2_, underscores the inherent trade-offs that dictate their appropriateness for supercapacitor applications. Carbon-based materials, including graphene, carbon nanotubes, and activated carbon, predominantly utilise electric double-layer capacitance (EDLC), which is advantageous due to its extensive surface area, excellent electrical conductivity, and remarkable cycling stability, but it exhibits modest specific capacitance and energy density. Transition metal oxides, including MnO_2_, NiO, and Co_3_O_4_, exhibit robust pseudocapacitive performance owing to rapid and reversible redox reactions. Specific capacitance values may exceed 1000 F g; nevertheless, they are sometimes constrained by low conductivity and cycling stability.^56,57^ Conducting polymers such as polyaniline (PANI), polypyrrole (PPy), and PEDOT exhibit significant pseudocapacitance while maintaining flexibility and processability; nonetheless, their long-term durability under repetitive cycling poses a challenge.^58^ Transition metal dichalcogenides (TMDs) such as MoS_2_ and WS_2_ demonstrate a hybrid charge storage mechanism that integrates electric double-layer capacitance (EDLC) with pseudocapacitance, attributable to their layered architecture, diverse phases, and abundant active sites.^59,60^ They are recognised as attractive alternatives for next-generation supercapacitor electrodes owing to their moderate-to-high capacitance and adjustable electrochemical properties. Metal oxides and layered double hydroxides (LDHs) provide exceptional pseudocapacitive performance and energy density; yet, they are constrained by limited conductivity and comparatively inadequate stability at elevated speeds. MXenes exhibit metallic conductivity and elevated capacitance; nonetheless, they encounter issues with restacking and oxidation. Black phosphorus has rapid ion transport and elevated pseudocapacitance, although it is deficient in environmental stability. In contrast, MoS_2_ and other two-dimensional transition metal dichalcogenides offer a harmonious blend of redox activity, adjustable electronic phases, and structural adaptability; still, enhancements in conductivity and cycling longevity, particularly for 2H-MoS_2_, are required. Table 2 highlights that no individual material satisfies all performance criteria, emphasising the necessity of hybrid and composite approaches to effectively combine conductivity, stability, energy density, and cost-efficiency.

**Table 2:** Comparative analysis of key electrode material families for supercapacitors

Material class	Specific capacitance (F g^−1^)	Rate capability	Cycling stability	Energy/power density	Cost/scalability	Key advantages	Key limitations	References
Carbon materials (AC, CNTs, graphene)	80–300	Excellent	>10 000 cycles	Moderate E, high P	Very low cost, scalable	High conductivity, stable	Limited capacitance (EDLC mechanism), graphene restacking	63 and 64
Metaloxides/hydroxides (MnO_2_, NiO, Co_3_O_4_)	300–1500	Moderate	Often poor at high rates	High E, moderate P	Moderate cost	Very high capacitance	Low conductivity, structural degradation	65 and 66
Conducting polymers (PANI, PPy, PEDOT)	300–800	Good	Poor–moderate	Moderate E & P	Low cost	High pseudocapacitance	Mechanical degradation during cycling	67 and 68
2D TMDs (MoS_2_, WS_2_, *etc.*)	<700 (1T phase >700)	Moderate	Good–moderate	High E, moderate P	Moderate	Tunable phase, pseudocapacitive	2H low conductivity; 1T instability	69 and 70
Graphene	100–300	Excellent	Excellent	High P, low E	Low cost	High conductivity	Non-faradaic → limited capacitance	71
MXenes (Ti_3_C_2_T_*x*_, *etc.*)	800–1500+	Excellent	Moderate	High E & P	Moderate–high	Metallic conductivity, redox activity	Oxidation, restacking	72
Black phosphorus (BP)	300–600	Good	Poor	High E	Low–moderate	Fast ion diffusion	Extremely unstable in the air	73
Layered double hydroxides (LDHs)	1000–1500	Moderate	Moderate	High E	Low cost	Abundant redox sites	Low conductivity, structural instability	74 and 75
Hybrid/composite materials	High	High	High	High	Moderate	Synergistic enhancement	More complex synthesis; higher cost	76 and 77

**Fig. 11:**
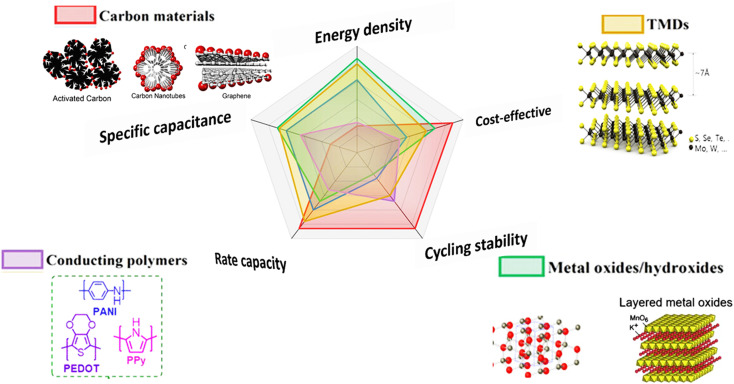
Diagrammatic illustration of key SC electrode materials based on their specific capacitance, rate capability, cycling stability, cost-effectiveness, and energy/power density Adapted from ref. 62 (*Small Methods*, 2023) with permission from John Wiley & Sons, copyright © 2023.

Fig. 11 presents a comparison of various electrode materials employed in supercapacitors, including factors such as specific capacitance, rate capability, cycling stability, cost-effectiveness, and energy density. Transition metal oxides demonstrate elevated specific capacities and energy densities, rendering them appealing for energy storage applications. Metal oxides surpass alternative materials for energy density and specific capacitance. Nonetheless, their practical implementation is occasionally constrained by inadequate cycling stability, particularly under high-rate charge and discharge settings. Carbon materials exhibit exceptional cost-effectiveness and cycling stability; yet, they display comparatively modest capacitance and energy density due to their non-faradaic double-layer storing mechanism.^61^ Conducting polymers have enough capacitance and rate capability; however, they encounter issues with long-term stability, mainly due to structural deterioration after repeated charge and discharge cycles. TMDs proficiently regulate various parameters, particularly energy density and pseudocapacitive behaviour; yet, improvements in rate capability and cycling longevity are still required. This comparison underscores the trade-offs inherent in choosing materials for certain supercapacitor applications and accentuates the importance of hybrid materials and composites in leveraging the advantages of various components.

Among various supercapacitor materials, 2D TMDs have recently gained significant attention owing to their distinctive layered structures, large surface areas, and superior electronic, optical, and electrochemical properties.^78–80^ These characteristics enable 2D TMDs to facilitate efficient ion intercalation and surface redox reactions, positioning them as exceptional contenders for top-tier performance HCs electrodes.

### 3.2 Two-dimensional transition metal dichalcogenides (2D TMDs)

**Fig. 12:**
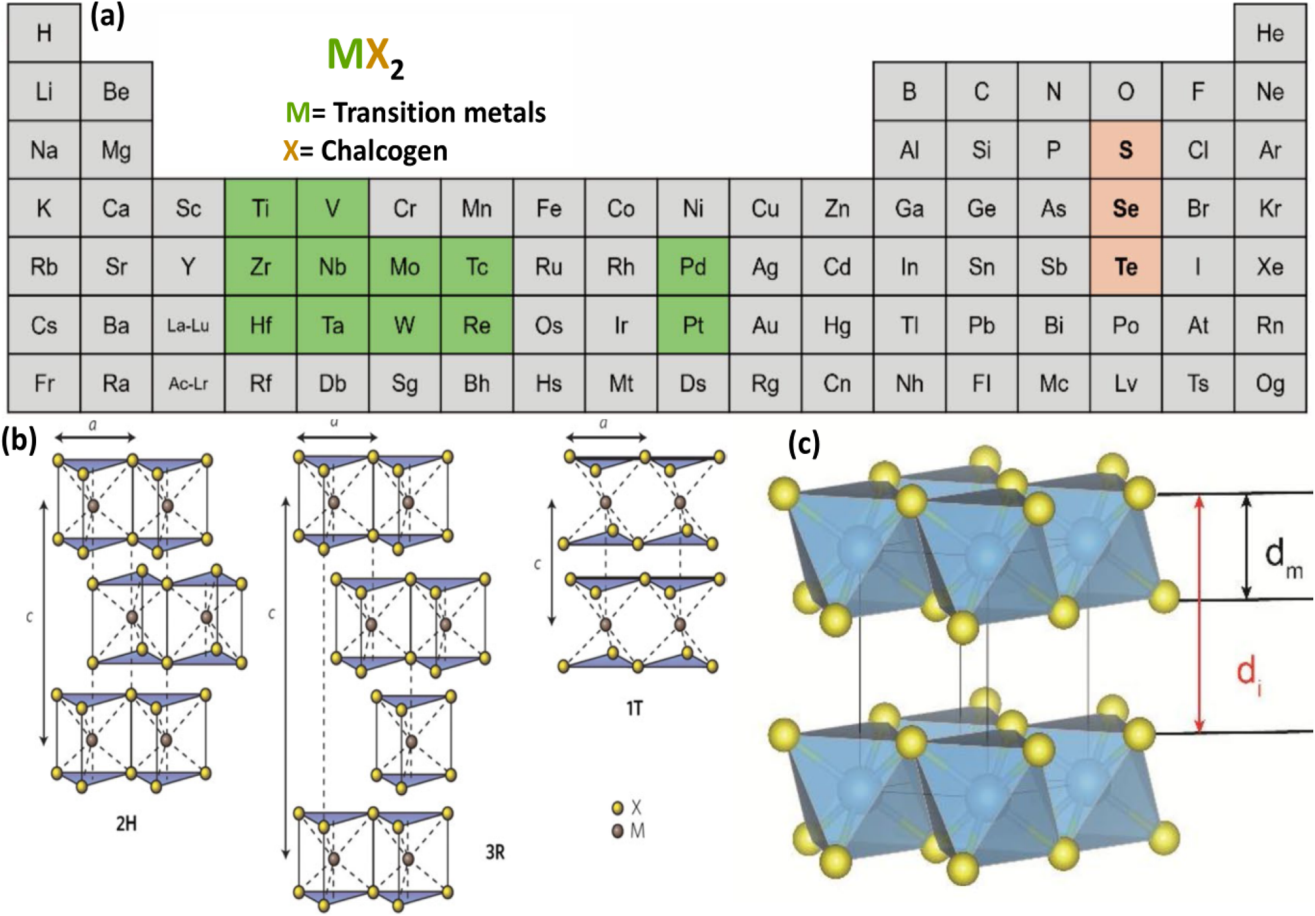
(a) Periodic table highlighting the elements used to form TMDs, (b) crystal structures of common TMD polytypes: 2H, 3R, and 1T, illustrating differences in stacking and coordination geometry^88^ (c) schematic of a layered TMD structure showing the monolayer thickness (*d*_m_) and interlayer distance (*d*_i_) separated by van der Waals gaps, reproduced from ref. 93 (*Scientific Reports*, 2025).

The remarkable properties of graphene-based materials have inspired researchers to investigate other 2D materials, especially TMDs.^81^ In 2004, the first graphene crystal known for its mechanical strength surpassing that of steel was successfully isolated.^82,83^ After this discovery, several 2D materials with different physical and chemical properties were separated. The formula MX_2_ is often used to show 2D TMD materials. In this formula, M stands for a transition metal like Mo, W, or Re, while X stands for a chalcogen element like S, Se, or Te Fig. 12a.^84–86^ These combinations form layered compounds with unique electronic and structural properties. WS_2_, MoS_2_, WSe_2_ and MoSe_2_ are common 2D TMDs materials.^87^

Fig. 12b shows that 2D TMD materials can occur in different crystalline phases: tetragonal (1T), hexagonal (2H), and rhombohedral (3R). The way the atoms are arranged and stacked in these phases is different, which has an effect on their electrical, optical, and mechanical properties. The 2H phase is the most stable and is available in semiconducting 2D TMDs. The 1T phase, on the other hand, is usually metallic and is usually metastable. It can be made stable with chemical or electrochemical treatments. The 3R phase is less common, but it has its own distinctive features because of how its layers stack on top of each other.^88^

In 2D TMD materials, layers are interconnected by weak van der Waals bonds, while the M and X are bonded through covalent interactions.^89–92^Fig. 12c provides a schematic of the layered 2D TMD structure, emphasizing the monolayer thickness (*d*_m_) and the interlayer distance (*d*_i_), which includes the van der Waals gap between adjacent layers.^93^ The inherently weak interactions between the layers allow these materials to be readily exfoliated into mono- or few-layer nanosheets. Such thinning greatly enhances their surface reactivity and mechanical flexibility, making them highly promising candidates for emerging nanoelectronic systems and advanced energy-storage technologies.^94^

#### 3.2.1 Classification of 2D TMDs based on their electronic and magnetic properties

The diverse behaviour of TMDs arises from the variation in M and X elements. Properties of 2D TMDs range from semiconductor (*i.e.*, MoS_2_ and WS_2_) to true metals (*i.e.*, NbS_2_ and VSe_2_), which vary significantly depending on the group and type of metal (M) and chalcogen (X) elements involved.^95,96^ Group 4 TMDs such as Ti, Hf, and Zr combined with S, Se, or Te exhibit semiconducting behaviour with band gaps ranging from 0.2 to 2 eV and are typically diamagnetic.^97^ Group 5 TMDs, including V, Nb, and Ta, display narrow band metallic or semimetallic properties with low electrical resistivity and high electronic conductivity.^98^ Group 6 TMDs, such as Mo and W, are widely studied for their semiconducting sulfides and selenides (*E*_g_ ∼1 eV), whereas their telluride counterparts tend to be semimetallic.^99^ Group 7 TMDs (Tc and Re-based) are typically small-gap semiconductors. Notably, PdTe_2_ exhibits superconductivity.^97^ This rich diversity of electronic characteristics among TMDs makes them versatile candidates for applications ranging from semiconductors to superconductors in electronic and energy storage devices. Table 3 summarizes the classification of 2D TMDs according to their group-wise electronic and magnetic properties.

**Table 3:** Classification of 2D TMDs based on their electronic

Group	Representative MX_2_		Electronic properties	Special features	References
	Metals (M)	Chalcogens (X)			
Group 4	Ti, Zr, Hf	S, Se, Te	Semiconductors	Layer-dependent band gap	97
Group 5	V, Nb, Ta	S, Se, Te	Semimetallic/metallic	High electronic conductivity	97
Group 6	Mo, W	S, Se	Semiconductors	Widely used in optoelectronics	99
	Mo, W	Te	Semimetallic	Phase tunable (2H ↔ 1T)	
Group 7	Re, Tc	S, Se, Te	Small-gap semiconductors	—	100
Group 10	Pd, Pt	S, Se	Semiconductors	—	97
	Pd, Pt	Te	Metallic	PdTe_2_ is superconducting	

The diverse behavior observed in TMDs stems not only from differences in the metal (M) and chalcogen (X) elements but also from the more intricate interplay of structural and compositional parameters. Their properties depend on the particular M–X combination, the structural phase they adopt (1T, 2H, or 3R), and even their dimensionality, whether in bulk form or as monolayers. These variations give rise to marked differences in both electronic and electrochemical characteristics. Moreover, synthesis-related imperfections, including point defects, vacancies, and irregularities at the edges, can substantially modify or even improve material performance.

Crucially, these factors rarely operate in isolation. Instead, they work together synergistically to shape conductivity, charge-storage behavior, surface reactivity, and ultimately the performance of the final device. The next section of this review examines how these interconnected parameters govern the suitability of MoS_2_-based TMDs for supercapacitor applications.

### 3.3 Structural and electronic properties of 2D MoS_2_

Among the family of TMDs, 2D MoS_2_ has attracted the greatest research interest due to its distinctive chemical and optical properties.^101,102^ Structurally, each Mo atoms in 2D MoS_2_ are positioned between two sulphur atoms, creating layers that are three atoms thick arrangement.^103^ Strong covalent bonding holds the atoms together within each layer, whereas adjacent layers are stacked through weaker van der Waals forces. The combination of a large accessible surface area and abundant coordination sites make 2D MoS_2_ highly favorable interfacial characteristics. These features promote efficient charge interaction at the electrode surface, supporting both faradaic and non-faradaic processes. As a result, 2D MoS_2_ is considered a promising material for a wide range of energy conversion and storage technologies.

**Fig. 13:**
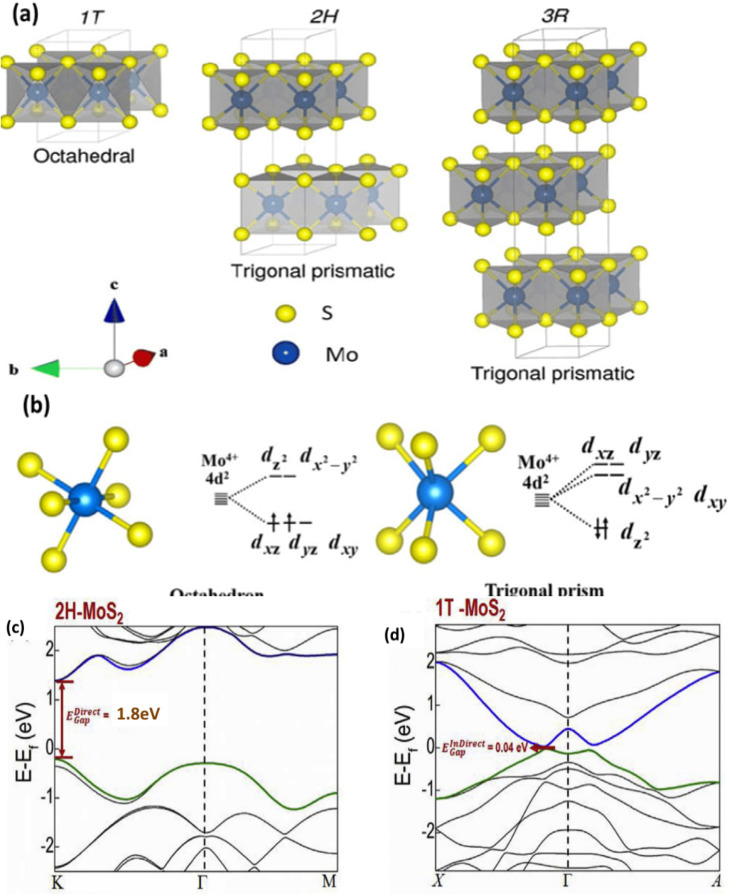
Crystal structures of 1T, 2H, and 3R MoS_2_ (a) adapted from ref. 109 (*Mater. Adv.*, 2022) with permission from the Royal Society of Chemistry, © 2022, d-orbital splitting diagram in trigonal prismatic and octahedral coordination (b),^108^ electronic band structures for 2H MoS_2_ (c) and 1T MoS_2_ (d) are adapted from ref. 111 (*Journal of Materiomics*, 2018) with permission from Elsevier, copyright © 2018.

Various polytypes of MoS_2_ (1T, 2H, and 3R) structures exist, characterised by distinct arrangements of Mo and S atoms.^104,105^ The 1T MoS_2_ structure has molybdenum atoms joined in an octahedral way by sulphur atoms to make a unit cell. This creates a metallic structure with an ABC stacking sequence. This configuration puts each new layer immediately on top of the one before it, without any symmetry in rotation. The 2H phase has a trigonal prismatic coordination and stacks in the order AbA-BaB-AbA, which gives it hexagonal symmetry and makes it act like a semiconductor. There are two S-Mo-S layers in each unit cell along the *c*-axis. The 3R phase similarly has trigonal prismatic coordination, but its stacking order is different, following the rhombohedral AbA-BcB-CaC sequence. There are three S-Mo-S layers in each unit cell of the rhombohedral 3R-polytype. The order in which 2H and 3R are stacked is different. 2H is stacked as AbA, BaB, AbA, and 3R is stacked as AbA, BcB, CaC, AbA.^106^ Additionally, the 3R phase exhibits metastability and can be readily converted to the 2H phase, which diminishes its competitive edge in energy storage applications when compared to the 2H phase.^107^Fig. 13a illustrates the structural differences among the three primary polymorphs of MoS_2_: 1T, 2H, and 3R, highlighting their atomic coordination and stacking arrangements.

These structural polymorphs have a pronounced impact on the physical behavior of MoS_2_. The 2H phase is the most thermodynamically stable and the form most commonly occurring in nature, while the 1T and 3R phases are metastable. Each structure possesses distinct atomic arrangements and electronic characteristics, which in turn determine its suitability for different technological applications.

Notably, the electronic properties of the 2H and 1T phases differ substantially. The 2H phase features trigonal prismatic coordination with AbA-BaB stacking and exhibits semiconducting behavior, making it particularly attractive for optoelectronic applications. In contrast, the 1T phase displays an octahedral coordination environment and metallic conductivity, which lends itself to electrochemical and catalytic uses.

Ouyang *et al.* studied the electrical properties of MoS_2_ in both its 2H and 1T phases and found that their band structures were very different. These changes are strongly related to the crystal structure of MoS_2_, which has a big effect on its electrical characteristics. According to crystal field theory, the 2H phase has trigonal prismatic coordination (TPC) around Mo^4+^ ions. This splits the 4d orbitals so that the d_*z*^2^_ orbital is filled and the d_*xy*_ and d_*x*^2^−y^2^_ orbitals stay empty, as seen in Fig. 13b.^108^ This orbital arrangement leads to a closed-shell configuration (4d^2^) and imparts semiconducting behaviour to the 2H-MoS_2_, which typically has a bandgap of approximately 1.8 eV and exhibits low electrical conductivity in its undoped state (Fig. 10c).

In contrast, the 1T phase displays octahedral coordination around Mo atoms, resulting in a different crystal field splitting where the 4d orbitals are divided into t_2_g (d_*xy*_, d_*xz*_, d_*yz*_) and eg (d_*z*^2^_, d_*x*^2^−y^2^_) subsets, as shown in Fig. 13b. In this configuration, the Mo^4+^ ions' two 4d electrons partially fill the t_2_g orbitals, contributing to delocalized electronic states and thus metallic conductivity. The 1T phase generally has a narrow bandgap and strongly depends on the functional group type and its coverage, and can be tuned from zero to ∼1 eV.^109,110^ However, the 1T phase is metastable and tends to revert to the more energetically favourable 2H phase unless chemically stabilized or hybridized. Its near-zero bandgap and high conductivity make it well-suited for roles in electrocatalysis and as a conductive component in energy storage systems.^111^

Moreover, sulphur atoms located at the edges of MoS_2_ layers possess lone pair electrons that contribute to edge passivation, enhancing the material's resistance to environmental degradation. The distinct molecular and electronic structures of the 2H and 1T phases greatly influence their behaviour in energy storage applications. While the 1T phase offers superior conductivity for rapid charge movement, the 2H phase ensures mechanical and chemical stability for long-term cycling. Therefore, strategies such as phase control, chemical intercalation, and composite formation are commonly adopted to fine-tune MoS_2_ properties for high-performance supercapacitor electrodes.

#### 3.3.1 Phase transition between 2H MoS_2_ and 1T MoS_2_

MoS_2_'s electronic characteristics can be tailored for numerous applications, including energy storage and catalysis, by phase engineering. As discussed earlier, MoS_2_ comes in three polymorphs, and the most common are semiconducting 2H and metallic 1T.^112^ These two phases have fundamentally different electrical structures and can be interconverted using intralayer atomic plane gliding, which is triggered by the transverse displacement of sulphur planes.^112^

The 1T phase of MoS_2_ exhibits significantly higher electrical conductivity compared to its semiconducting 2H counterpart, making it highly desirable for applications in electrocatalysis, energy storage electrodes, and conductive composites. To exploit the advantageous properties of different MoS_2_ phases, several strategies have been developed to induce and stabilize the transition from the semiconducting 2H phase to the metallic 1T phase. A widely used method is alkali-metal intercalation, typically with lithium (Li) or potassium (K) ions, which introduces charge transfer and lattice distortion, thereby driving the material into the 1T configuration. The metallic phase can be further stabilized through substitutional doping with electron-rich elements such as rhenium (Re), technetium (Tc), or manganese (Mn). These dopants help preserve the distorted octahedral coordination that characterizes the 1T phase.

Park *et al.* introduced a molten-metal-assisted intercalation (MMI) technique to synthesize high-purity 1T-phase TMDs. In their approach, molten potassium reacts with MoS_2_ to form K–S ionic bonds, facilitating electron transfer to the Mo centers. This electron redistribution alters the Mo d-orbital states and effectively stabilizes the 1T structure. Using this method, the authors achieved a phase purity exceeding 92%.^113^ The effective intercalation of potassium into MoS_2_ using the MMI method is primarily governed by their electronic characteristics. Because the electron affinity of MoS_2_ (4.45 eV) is slightly higher than the ionization potential of potassium (4.34 eV), electrons can spontaneously transfer from K to the MoS_2_ lattice, enabling and stabilizing the intercalation process.^113^ The low ionisation energy of K is readily available to donate electrons, while MoS_2_, having a high electron affinity, efficiently accepts them. Additionally, potassium atoms form K–S ionic bonds due to the higher electronegativity of sulphur compared to molybdenum. The significant electronegativity difference between potassium and sulphur further promotes electron transfer and bonding.^114^ This effective intercalation and doping mechanism not only stabilizes the 1T phase but also enables the scalable synthesis of high-purity 1T-MoS_2_ powders *via* the MMI approach.

**Fig. 14:**
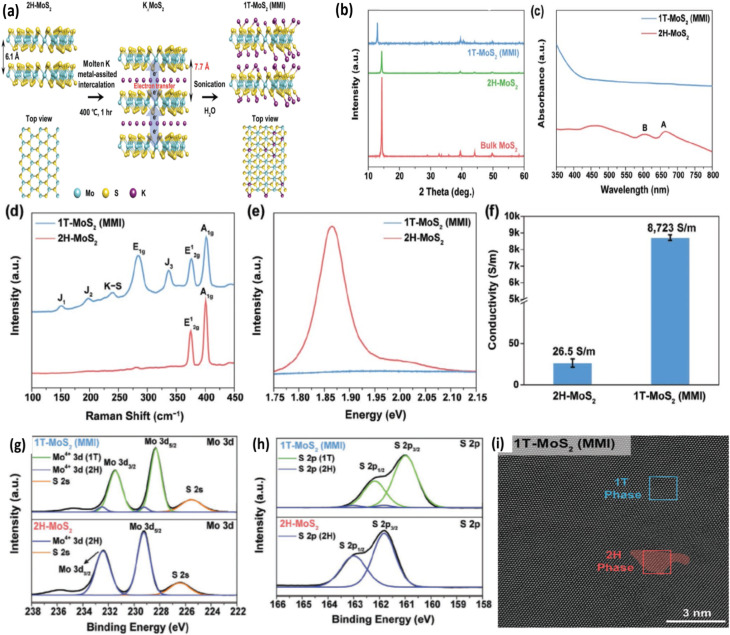
(a) Representation conversion of 2H-MoS_2_ to 1T-MoS_2_, (b) comparison of XRD spectra of 1T-MoS_2_ (MMI), 2H-MoS_2_ nanosheets, and bulk MoS_2_, (c) UV-Vis–NIR spectra of 1T-MoS_2_ (MMI) and 2H-MoS_2_, (d) Raman spectra of 1T-MoS_2_ (MMI) and 2H-MoS_2_, (e) PL spectra of 1T-MoS_2_ (MMI) and 2H-MoS_2_, demonstrating the enhanced charge transfer in metallic 1T-MoS_2_, (f) comparison of electrical conductivities between 1T-MoS_2_ (MMI) and 2H-MoS_2_ nanosheets, (g and h) XPS spectra of 1T-MoS_2_ (MMI) and 2H-MoS_2_, showing the Mo 3d, S 2p, and (i) HRTEM image of intercalated 1T-MoS_2_, zoomed images of two phases (2H and 1T phase), reproduced from ref. 113 (*Advanced Materials*, 2020) with permission from John Wiley & Sons, copyright © 2020.

The intercalation of potassium, its resulting doping effect on the MoS_2_ basal plane, and the exfoliation of 1T-MoS_2_ were first confirmed using X-ray diffraction (XRD), UV-Vis–NIR spectroscopy, Raman spectroscopy, photoluminescence (PL), X-ray photoelectron spectroscopy (XPS), and high-resolution transmission electron microscopy (HRTEM) Fig. 14. Fig. 14b shows the XRD pattern of potassium-intercalated MoS_2_ (K_*x*_MoS_2_). The preferred (002) reflection of intercalated MoS_2_ appears at 11.4°, whereas bulk 2H-MoS_2_ shows the (002) peak at 14.4°. The blueshift of the (002) peak in 1T-MoS_2_ (MMI) compared with 2H-MoS_2_ nanosheets and bulk MoS_2_ indicates successful intercalation of reactive molten potassium into the MoS_2_ interlayers and effective electron doping of the MoS_2_ basal plane. As shown in Fig. 14c, 2H-MoS_2_ exhibits the typical semiconducting A and B excitonic peaks at 664 nm and 601 nm, corresponding to direct transitions from the valence to conduction band. In contrast, 1T-MoS_2_ (MMI) shows a broad, featureless absorption spectrum with suppressed excitonic peaks, confirming conversion of the semiconducting 2H phase to the metallic 1T phase.

Raman spectroscopy, as depicted in Fig. 14d, demonstrates the characteristic vibrational modes of the 1T phase alongside unique K–S bonding signals, further verifying successful intercalation. PL analysis (Fig. 14e) shows a pronounced emission peak at 1.87 eV for 2H-MoS_2_, associated with its direct optical bandgap. This emission is absent in 1T-MoS_2_ because rapid charge-carrier relaxation and strong metallic screening suppress the photoluminescence signal, providing further evidence of its metallic nature. As shown in Fig. 11e, the electrical conductivity of MMI-derived 1T-MoS_2_ is markedly higher than that of the semiconducting 2H phase, reinforcing both its metallic character and its suitability for high-performance electrochemical applications. The phase purity and the influence of potassium intercalation were further evaluated using X-ray photoelectron spectroscopy (XPS). For 2H-MoS_2_, the Mo 3d_5/2_ and 3d_3/2_ peaks appear at 229.1 eV and 232.2 eV, respectively. In 1T-MoS_2_ (MMI), these peaks shift downward to 228.2 eV and 231.3 eV (Fig. 14g). A similar downshift is seen in the S 2p_3/2_ and S 2p_1/2_ peaks: from 161.9 eV and 163.1 eV in 2H-MoS_2_ to 161.0 eV and 162.2 eV in 1T-MoS_2_ (Fig. 14h). These binding-energy shifts arise from Fermi-level movement due to electron filling of Mo d-orbitals, confirming electron donation from potassium atoms during the MMI process. XPS quantification reveals a phase composition of 92.3% 1T and 7.7% 2H, demonstrating that the MMI approach yields MoS_2_ flakes with high 1T-phase purity.^113^

The HRTEM image in Fig. 14i reveals dominant regions of the 1T phase with minor traces of the 2H phase; the zoomed-in lattice images distinctly show octahedral coordination in the 1T structure and TPC in the 2H counterpart.

Photochemical techniques have also been employed, where light exposure promotes electronic rearrangement, triggering the phase change. This technique presents a promising alternative to traditional alkali metal intercalation methods, which often involve hazardous reagents such as *n*-butyllithium and require inert atmospheric conditions, making them time-consuming, unsafe, and expensive. In contrast, the photochemical method is a benign, rapid, and scalable approach. Under light irradiation, monolayer 2H-MoS_2_ absorbs photons, generating electron–hole pairs.

**Fig. 15:**
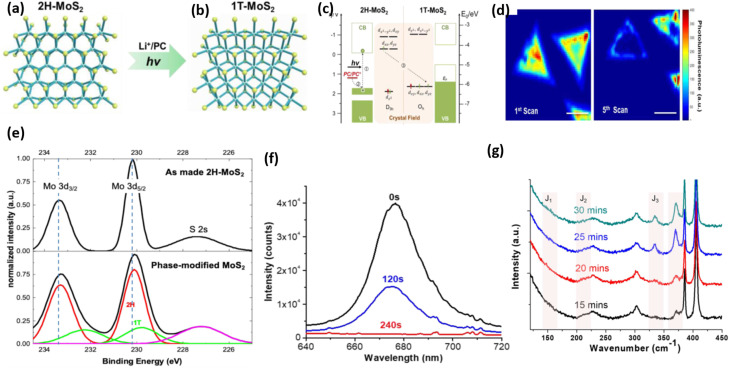
Representation of 2H-to-1T phase conversion *via* Li^+^/PC and light (a and b), band structure evolution showing semiconducting 2H and metallic 1T configurations (c), Photoluminescence (PL) maps showing PL quenching after repeated scans (d), XPS spectra confirming Mo 3d peak shifts upon phase transition (e), Time-dependent PL spectra showing decreased intensity with exposure time (f), Raman spectra following the time evolution of a monolayer MoS_2_ flake during photochemical phase change (g), adapted from ref. 115 (*Frontiers in Chemistry*, 2019).

Byrley *et al.* introduced a photochemical approach to initiate a semiconductor-to-metal phase change in monolayer MoS_2_ under mild chemical conditions.^115^ Their investigation revealed that photoinduced electrons, generated through band-gap excitation, carry enough chemical potential to cause phase conversion when an electron-donating solvent is present. Control experiments underscored the significance of the redox environment in the process.^115^Fig. 12 shows the systematic transition of 2H-MoS_2_ to metallic 1T-MoS_2_; the structural transformation caused by lithium-ion intercalation under photon irradiation is illustrated in Fig. 15a and b, highlighting the transition. The band structure diagrams presented (Fig. 15c) illustrate the electronic consequences of this transition.

In 2H-MoS_2_, the valence and conduction bands are separated by a well-defined bandgap, whereas in the 1T phase, the Fermi level lies within the Mo d-orbitals, reflecting its metallic character. The PL mapping results further support the loss of semiconducting behavior, as the photoluminescence intensity decreases markedly after repeated scans. This trend is also evident in the time-dependent PL spectra (Fig. 15f), where the emission signal progressively weakens with longer exposure, indicating a gradual phase transition. X-ray photoelectron spectroscopy (XPS) measurements provide complementary confirmation of the photochemically induced 2H → 1T phase transformation, consistent with the Raman observations. The XPS analysis (Fig. 15e) reveals new Mo 3d peaks associated with the 1T phase, confirming the phase change at the atomic level.

Fig. 15g shows the time-resolved Raman spectra of a monolayer MoS_2_ flake under 532 nm laser illumination (0.14 mW µm^−2^). Two new peaks at 330 cm^−1^ and 370 cm^−1^, characteristic of the emerging 1T phase, gradually appear with increasing illumination time. The frequency separation of 19.8 cm^−1^ between the E^1^_2_g and A_1g_ modes confirms that the flake is a monolayer. The Raman spectrum exhibits the typical 2H-MoS_2_ phonon modes at 382 cm^−1^ (E^1^_2_g) and 402 cm^−1^ (A_1g_). For chemically exfoliated 1T-MoS_2_ prepared using *n*-butyllithium, three well-known superlattice peaks 150 cm^−1^ (*J*_1_), 226 cm^−1^ (*J*_2_), and 333 cm^−1^ (*J*_3_) are typically observed, as reported by Zhu *et al.* (2017), and are widely used as Raman fingerprints of the 1T phase. In the present study, the lowest-frequency *J*_1_ peak is too weak to be distinguished from the residual laser background below 175 cm^−1^. The *J*_2_ feature overlaps with the LA(M) mode of MoS_2_ near 227 cm^−1^, making it difficult to resolve. However, with increasing illumination time, a distinct *J*_3_ peak (∼333 cm^−1^) becomes evident after ∼20 min under 0.14 mW µm^−2^ laser power, providing clear Raman evidence for the formation of the 1T phase. In addition, the emergence of the ∼370 cm^−1^ peak also previously observed in chemically exfoliated 1T-MoS_2,_ further supports the structural transition. Overall, these results provide strong evidence from multiple techniques for the effective and controlled phase transition in MoS_2_.

Intercalating ions or molecules such as lithium can induce a phase transition in MoS_2_ from 2H to 1T, enhancing conductivity and pseudocapacitance.^116^ However, this strategy is often complex and dangerous. The hydrothermal process, on the other hand, is a simpler and more environmentally responsible way to make 1T-MoS_2_. These improvements together make it possible to change the properties of MoS_2_ in a lot of different ways.^117^ Typically, MoS_2_ produced by hydrothermal synthesis consists of a 2H/1T hybrid phase, with the 1T phase stabilized by the surrounding 2H structure. Despite its advantages, producing few-layer or monolayer MoS_2_ through hydrothermal synthesis remains difficult, which limits its applicability in high-performance electrode systems. To overcome this limitation, combining MoS_2_ with complementary materials, particularly conducting polymers, has proven an effective strategy for enhancing its electrochemical behavior. For example, Tian *et al.* prepared a MoS_2_/PPy hybrid by first synthesizing MoS_2_ hydrothermally and then electrodepositing ultrathin PPy films *via* potential scanning.^122^ The intercalation of PPy not only promoted monolayer formation but also triggered a 2H → 1T phase transition, resulting in markedly improved electrochemical performance compared with pristine MoS_2_ and pure PPy.

**Fig. 16:**
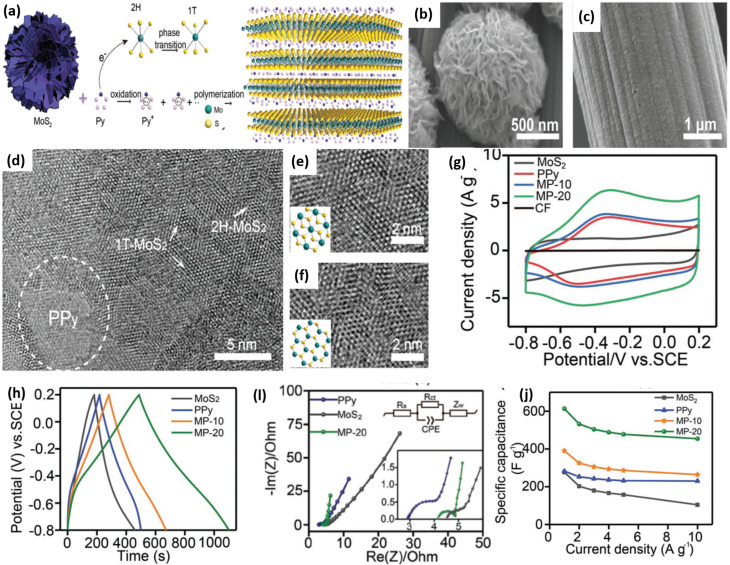
Schematic illustration of the formation of 1T-phase monolayer MoS_2_*via* PPy anchoring through oxidation and *in situ* polymerization of pyrrole (Py), inducing a 2H-to-1T phase transition (a), SEM images of pristine MoS_2_ (b), pure PPy (c), HRTEM image of MP-20 showing the coexistence of 1T and 2H phases (d), High-resolution images displaying 1T-MoS_2_ (e) and 2H-MoS_2_ lattice structures (f), CV curves showing enhanced capacitive behavior of MP-20 (g), GCD profiles at 1 A g^−1^ (h), Nyquist plots demonstrating lower charge-transfer resistance for MP-20; inset: zoomed-in high-frequency region and equivalent circuit model (i), specific capacitance *vs.* current density comparison, highlighting the superior performance of MP-20 (j), reproduced from ref. 118 (*Advanced Materials Interfaces*, 2019) with permission from John Wiley & Sons, copyright © 2019.

Conducting polymers such as PPy facilitate this phase transition by injecting electrons into MoS_2_ during polymerization. Fig. 16 summarizes the synthesis process and structural evolution of monolayer 1T-MoS_2_ nanosheets anchored with polypyrrole. The schematic in Fig. 16a illustrates how electron transfer during pyrrole oxidation drives the 2H-to-1T conversion. SEM images (Fig. 16b–d) show the progressive morphological changes from pristine MoS_2_ to pure PPy and finally to the MoS_2_/PPy hybrid. The HRTEM image (Fig. 16d) clearly displays the coexistence of both 1T and 2H phases in the MP-20 sample (the nanocomposite synthesized at a scan rate of 20 mV s^−1^), while the high-resolution images in Fig. 16e and f distinctly reveal the lattice structures associated with each phase.

Cyclic voltammetry curves (Fig. 16g) showed a significantly larger integrated area for MP-20, indicating enhanced capacitive behaviour. Galvanostatic charge–discharge (GCD) profiles (Fig. 16h) and Nyquist plots (Fig. 16i) demonstrated the composite's superior charge storage and lower resistance, respectively. The specific capacitance analysis (Fig. 16j) revealed that MP-20 retained higher capacitance even at increased current densities, confirming its excellent rate capability and potential as a high-performance electrode material.^118^

#### 3.3.2 Bulk *vs.* monolayer 2D MoS_2_

**Fig. 17:**
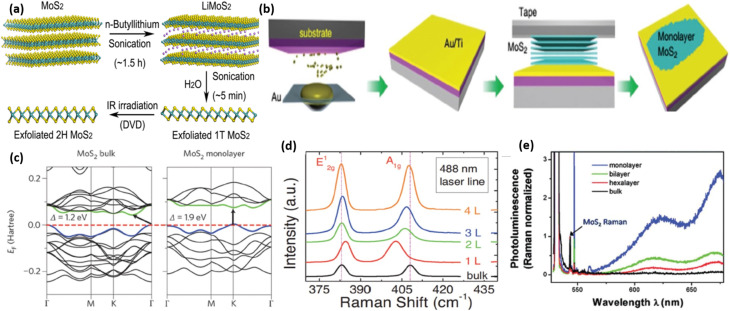
(a) chemical exfoliation of MoS_2_ using *n*-butyllithium under sonication,^119^ (b) Au-assisted exfoliation process of MoS_2_ (ref. 124) (c) electronic band structures of both bulk and monolayer MoS_2_, (d) Raman spectra were recorded from different regions of the sample with varying layer thicknesses. (e) Photoluminescence measurements, adjusted using Raman intensity for MoS_2_ layers of different thicknesses, revealed a significant enhancement in light emission efficiency in the monolayer form, reproduced from ref. 121 (*Nanoscale*, 2015) with permission from Royal Society of Chemistry, 2015.

2D molybdenum disulfide exhibits distinct physical, chemical, and electronic properties depending on its thickness. Bulk MoS_2_ comprises several layers interconnected by weak van der Waals forces, with each layer containing a molybdenum atom located between two sulphur atoms in an S-Mo-S arrangement, as depicted in Fig. 17a.^119^ Exfoliation of MoS_2_ into a single layer transforms it from an indirect to a direct bandgap semiconductor, which changes its electrical properties. In its bulk or multilayered form, MoS_2_ exhibits an indirect bandgap of approximately 1.2 eV, as illustrated in Fig. 17c.^120,121^ In contrast, monolayer MoS_2_ exhibits a direct bandgap nature, with its bandgap increasing to approximately 1.8–1.9 eV, as presented in Fig. 17c (right).^122,123^ This shift significantly enhances its optical absorption and photoluminescence, making monolayer MoS_2_ highly attractive for optoelectronic and sensing applications.

Furthermore, the conversion of bulk MoS_2_ material into a layered form considerably modifies its characteristics for energy storage applications. Firstly, monolayer 2D MoS_2_ provides a greatly increased surface area due to the complete exposure of its surface atoms, which promotes improved charge accumulation and rapid ion transport.^125^ Secondly, the material's numerous chemically active edge sites, more reactive than the relatively inert basal planes, along with open van der Waals gaps, support the effective intercalation of electrolyte ions.^97,126^ Additionally, MoS_2_ is characterised by its exceptional mechanical endurance and flexibility at the atomic scale.^125^

**Fig. 18:**
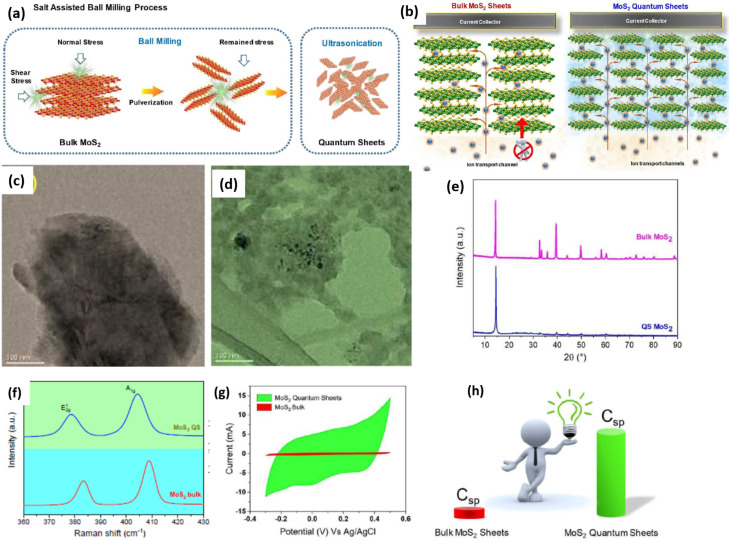
(a) Diagram showing the synthesis of MoS_2_ quantum sheets (QSs); (b) illustration depicting the charge storage mechanisms in both bulk MoS_2_ and MoS_2_ QSs, HRTEM images of (c) bulk MoS_2_, (d) MoS_2_ QSs, (e) XRD patterns showing a sharp contrast in crystallinity between bulk MoS_2_ and QSs, (f) Raman spectra of bulk MoS_2_ and MoS_2_ quantum sheets (QSs), along with (g) a comparison of their CV curves, Panel (h) illustrates the variation in specific capacitance between the bulk MoS_2_ and MoS_2_ QSs Reproduced from ref. 128 (*Chem. A*, 2020) with permission from the Royal Society of Chemistry.

Researchers have explored the influence of size on the physical and electrochemical behaviours of MoS_2_ through systematic investigations, including nanosheets, microsheets, spherical, flower-like structures, nanoribbons, nanotubes, and flake-like forms.^127^ MoS_2_ quantum dots (QDs) and quantum sheets (QSs) have appeared as we have continued to shrink things down to the quantum level. These materials look very promising for storing electrochemical energy. Nardekar *et al.* (2020) showed that QSs MoS_2_ (5–10 nm) made on a gramme scale utilising a salt-assisted ball milling process can hold more charge. Fig. 18a shows that this top–down strategy can make QSs that are only a few nanometres thick from bulk MoS_2_. This changes their physicochemical properties a lot. The morphological alteration is clearly shown in the HRTEM pictures (Fig. 18c and d), and the XRD and Raman studies (Fig. 18e and f) support the decrease in crystallinity and the changes in vibrational modes, respectively. These modifications directly impact the charge storage behavior, as depicted in Fig. 18b. Unlike bulk MoS_2_, where limited surface area and diffusion pathways restrict ion accessibility, QSs enable faster ion transport and enhanced interfacial interaction.

Electrochemical analysis revealed that MoS_2_ QSs exhibited superior capacitive behaviour compared to bulk MoS_2_, ascribed to the synergistic impact of quantum and electrochemical capacitance. Cyclic voltammetry (Fig. 18g) and specific capacitance measurements (Fig. 18h) further demonstrate that MoS_2_ QSs exhibit significantly improved electrochemical performance, validating their potential as advanced materials for high-efficiency SCs. Flexible symmetric supercapacitors (SSCs) constructed using MoS_2_ QSs delivered high specific capacitance (162 F g^−1^), energy density (14.4 Wh kg^−1^), excellent rate capability, and long cycle life, outperforming conventional MoS_2_-based supercapacitors.^128^

#### 3.3.3 Electrochemical energy storage applications of monolayer 2D MoS_2_

Molybdenum disulfide stands out as a highly investigated TMD for electrochemical energy storage applications, exhibiting a distinctive and diverse array of properties. The investigations into MoS_2_ cover beyond energy storage, including electronic sensors, biomedical engineering, *etc*.^129^ MoS_2_ possesses a structure similar to graphene and offers numerous benefits in electrochemical investigations.^130^ Additionally, the active Mo edges facilitate redox reactions, contributing extra capacitance *via* pseudocapacitive processes. The initial report regarding the application of MoS_2_ in supercapacitors emerged from the research conducted by Soon and Loh in 2007, highlighting the edge-oriented MoS_2_ film as a highly effective electrode material for supercapacitors.^131^ The edge-oriented structure offered an increased number of Mo sites for redox reactions.^132^

MoS_2_ stores charge through two primary mechanisms: (a) interlayer electrostatic charge accumulation, where electrolyte ions are confined within the stacked van der Waals gaps, and (b) faradaic processes involving redox activity localized at the molybdenum centres.^133^ During electrochemical cycling, ions can be inserted into the layered MoS_2_ structure.^134^ Analysis of the CV curves revealed prominent reduction peaks during the cathodic sweep, associated with the redox activity of molybdenum atoms at the edges of the MoS_2_ nanowalls.

Wang *et al.* prepared 2D MoS_2_ nanosheets that exhibited excellent electrochemical performance, delivering a specific capacitance of 143.12 F g^−1^ at a current density of 1.0 A g^−1^.^135^ This material uses a combination of EDLCs and a PC storing mechanism. The EDLC contribution arises from the physical adsorption and desorption of electrolyte ions on the electrode surface, forming a double-layer without electron transfer, as evidenced in CV curves, rectangular shape.^136^ In the meantime, the pseudocapacitive contribution stems from reversible faradaic reactions involving ion intercalation into MoS_2_ interlayers. The layered nature of MoS_2_, coupled with the induced sulphur vacancies, enhances ion transport and increases the density of electrochemically active sites. The charge storage behavior of MoS_2_ in Na^+^-based electrolytes involves both EDLC-type surface adsorption of Na^+^ ions (non-faradaic process), as shown in eqn (10), and pseudocapacitive intercalation of Na^+^ ions (faradaic process), as shown in eqn (11).

10 MoS_2_|Na^+^ ⇌ (MoS_2_·Na^+^)surface

11 MoS_2_ + *x*Na^+^ + *x*e ⇌ Na*_x_*MoS_2_

**Fig. 19:**
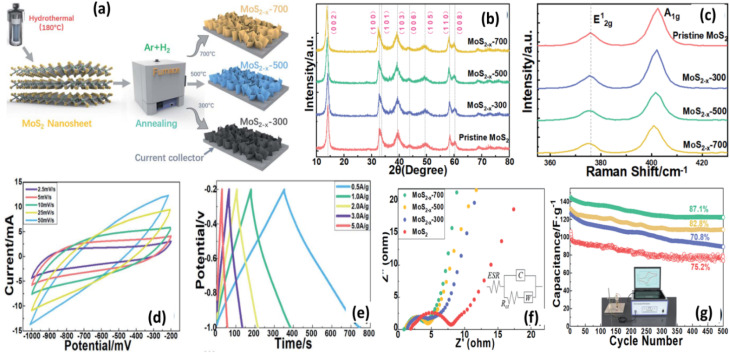
Diagrammatic presentation of the synthesis process of MoS_2−*x*_ nanosheets annealed at different temperatures (a), XRD (b), and Raman (c), CV curves (d), GCD profiles (e), and EIS spectra (f) reveal superior electrochemical behaviour of MoS_2−*x*_-700, Cycling performance (g) shows high stability with 87.1% after 5000 cycles, reproduced from ref. 135 (*RSC Advances*, 2021).

The CV curves (Fig. 19d) show a quasi-rectangular shape, indicative of a combination of EDL and PC characteristics. The GCD curves (Fig. 19e) show nearly symmetrical triangular profiles across various current densities, reflecting good capacitive reversibility. EIS (Fig. 19f) further demonstrates the lowest charge transfer resistance for MoS_2−*x*_-700, indicating enhanced ionic mobility and improved electrical conductivity.

The electrochemical behaviour of monolayer 2D MoS_2_ is heavily influenced by its morphology, exposed active sites, and interactions with electrolyte ions. DFT studies show that monolayer MoS_2_ exhibits significantly lower ion-diffusion barriers than multilayer structures due to shorter migration pathways and fully accessible basal planes. The metallic 1T phase further enhances charge-transfer kinetics through its high density of states at the Fermi level, enabling more efficient ion adsorption. Structural features such as expanded interlayer spacing (0.9–1.2 nm), edge-rich nanoflakes, and vertically aligned nanosheets have also been predicted to promote rapid ion intercalation. Recent investigations highlight that defect engineering markedly tunes the intrinsic electronic structure of MoS_2_. DFT calculations reveal that sulfur vacancies on the basal plane, Mo-edge, and S-edge introduce additional empty states near the Fermi level and create unpaired electrons, resulting in higher carrier density, improved conductivity, and extra active sites for ion adsorption. These electronic modifications, combined with the interconnected nanosheet morphology, account for the enhanced charge-storage behaviour and support the theoretical prediction that vacancy engineering significantly boosts the electrochemical performance of 2H-MoS_2_.^135^

**Fig. 20:**
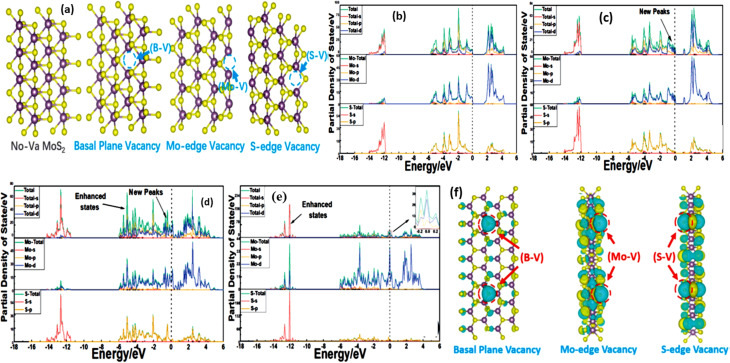
(a) Models of pristine MoS_2_ and MoS_2−*x*_ with basal-plane (B-V), Mo-edge (Mo-V), and S-edge (S-V) vacancies; (b–e) PDOS plots showing new Mo-4d states near the Fermi level and enhanced conductivity induced by different vacancy types, and (f) differential charge-density maps illustrating charge redistribution around vacancies, with S-edge defects showing the strongest electronic enhancement reproduced from ref. 135 (RSC Advances, 2021).

To understand the effect of sulfur vacancies on the electronic and electrochemical behaviour of MoS_2_, three vacancy configurations were examined: basal-plane vacancy (B-V), Mo-edge vacancy (Mo-V), and S-edge vacancy (S-V) (Fig. 20a). The PDOS of pristine monolayer MoS_2_ (Fig. 20b) shows a typical semiconducting character, with the VBM dominated by Mo-4d/S-3p hybridization and the CBM primarily composed of Mo-4d states, and no states crossing the Fermi level. Introduction of a basal-plane vacancy (Fig. 20c) creates new Mo-4d states near the Fermi level (−0.4–0 eV), indicating increased electronic activity and improved charge-transport capability. For the Mo–V defect (Fig. 20d), continuous electronic states appear across the Fermi level, confirming metallic behaviour. This occurs because edge Mo atoms become under-coordinated, generating additional empty 4d states and enhancing charge mobility. The S–V defect (Fig. 20e) shows an even stronger impact, introducing pronounced Mo-4d states near the Fermi level (−0.3 to 0.3 eV) and producing clear spin asymmetry characteristic of unpaired electrons. These unpaired carriers increase electrical conductivity, facilitate rapid charge migration, and contribute most significantly to the improved capacitance. The differential charge-density map (Fig. 20f) further verifies substantial charge redistribution at vacancy sites: charge depletion around S vacancies and accumulation on adjacent Mo atoms indicate the formation of dangling bonds and interstitial states that support efficient electron transport. Overall, S–V defects yield the largest enhancement in conductivity and electrochemical activity.

Moreover, interactions between the electrolyte and electrode are crucial. Protic electrolytes like H_2_SO_4_, KCl, Na_2_SO_4_, and KOH facilitate rapid H^+^ incorporation by reversible redox transitions (Mo^4+^/Mo^6+^). Recent experimental studies further illustrate the critical influence of shape and electrolyte on the electrochemical properties of MoS_2_. In an example work, conductive 1T-MoS_2_ nanosheets were self-assembled into nanoflower structures *via* a one-pot hydrothermal technique. The interconnected nanosheets in the flower-like configuration markedly enhance structural stability, electron transport routes, and electrolyte accessibility relative to isolated nanosheets.^137^ The nanoflower electrodes have significantly enhanced capacitance performance in both KCl (EDLC behaviour) and KOH (battery-type behaviour), as illustrated in Fig. 20h and k. The nanoflower structure in KCl has a capacitance of 483 F g^−1^ at 0.5 A g^−1^ and 305 F g^−1^ at 20 A g^−1^, significantly surpassing the nanosheet electrode, which shows 169 F g^−1^ and 120 F g^−1^, respectively. The structure exhibits 94–96% capacitance retention after 2000 cycles, demonstrating remarkable cycling stability. In KOH, the specific capacitance rises to 1120 F g^−1^ at 0.5 A g^−1^, highlighting the robust interaction between the hierarchical MoS_2_ structure and electrolyte ions.

**Fig. 21:**
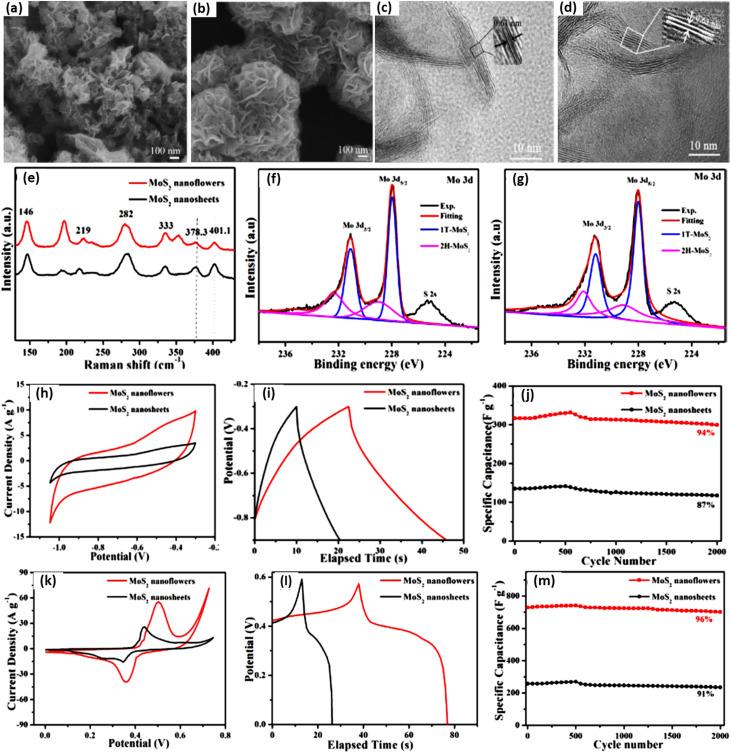
FESEM images of MoS_2_ nanosheets (a) and MoS_2_ nanoflowers (b); TEM images of MoS_2_ nanosheets (c) and MoS_2_ nanoflowers (d); Raman spectra of MoS_2_ nanoflowers and MoS_2_ nanosheets (e); XPS images from Mo 3d region of MoS_2_ nanoflowers (f); XPS images from Mo 3d region of MoS_2_ nanosheets (g); CV curves of MoS_2_ nanoflowers and MoS_2_ nanosheets at 20 mV s^−1^ in KCl (h); GCD curves of MoS_2_ nanoflowers and MoS_2_ nanosheets at 10 A g^−1^in KCl (i); cycling performance of MoS_2_ nanoflower and MoS_2_ nanosheet electrodes measured at 10 A g^−1^ for 2000 cycles in KCl (j); CV curves of MoS_2_ nanoflowers and MoS_2_ nanosheets at 20 mV s^−1^ in KOH (k); GCD curves of MoS_2_ nanoflowers and MoS_2_ nanosheets at 10 A g^−1^ in KOH (l); and cycling performance of MoS_2_ nanoflower and MoS_2_ nanosheet electrodes measured at 10 A g^−1^ for 2000 cycles in KOH (m) reproduced from, ref. 137 with permission from the American Chemical Society, © 2019, published under CC-BY-NC-ND 4.0.

The experiments in this study clearly demonstrate that assembling MoS_2_ nanosheets into a stable, interconnected nanoflower architecture greatly enhances electron transport, structural integrity, and the accessibility of electroactive sites. Furthermore, the electrolyte plays a decisive role: in KCl, the nanoflowers operate mainly through EDLC behaviour and deliver moderate capacitance, whereas in KOH, the strong faradaic interaction between OH^−^/K^+^ ions and MoS_2_ activates additional redox sites, resulting in a dramatically higher specific capacitance of up to 1120 F g^−1^ (Fig. 21).

Although 2D MoS_2_ monolayers possess attractive features, such as large active surface area, abundant edge sites, and short ion-diffusion paths, their practical application in supercapacitors is limited by intrinsic structural and electronic drawbacks. The metallic 1T phase, known for having high conductivity and almost negligible bandgap, is helpful for swift charge transport; nonetheless, it is metastable and frequently transitions back to the semiconducting 2H phase in the absence of stabilization. The 2H phase offers chemical stability and mechanical strength but is characterised by low electrical conductivity, chemically inert basal planes, and limited interlayer spacing, which hinder ion diffusion and intercalation kinetics.^138^ Additionally, the restacking of MoS_2_ nanosheets during electrode production prevents ion channels, restricts electrolyte availability, and diminishes cyclic stability. Consequently, 2H MoS_2_ electrodes frequently have reduced specific capacitance and restricted energy density relative to alternative advanced electrode materials. An effective way to enhance the performance of this material includes phase modulation and composite preparation to optimise MoS_2_'s electronic structure and enhance electrochemical characteristics.^139^

Combining MoS_2_ with conductive carbons (graphene, CNTs), transition metal oxides, sulfides, selenides, or conductive polymers has been widely employed to overcome its low conductivity, restacking tendency, and limited active sites. For example, a study conducted by Sarkar *et al.* (2013) a hybrid electrode consisting of MoS_2_ and rGO synthesized *via* a one-step hydrothermal method, where ammonia intercalation expanded the MoS_2_ interlayer spacing to 0.95 nm, while rGO enhanced conductivity.^139^ This architecture enabled faster Na^+^ intercalation/deintercalation, resulting in superior capacitance, rate capability, and cycling stability compared to pristine MoS_2_.

The synthesis of composites consisting of MoS_2_, metal oxides, and carbon nanotubes (CNTs) presents significant potential for enhancing supercapacitor technology. The combination of MoS_2_ and CNTs offers excellent conductivity and a large surface area, facilitating charge storage through both pseudocapacitive behaviour (intercalation) and EDLC mechanisms.^140^ As confirmed by XPS, the MoS_2_/CNT composite exhibited a lower binding energy shift for both Mo 3d and S 2p states compared to pristine MoS_2_.^141^ This alteration indicates a partial 2H → 1T phase transition, which is attributed to the transverse gliding of the S-plane during the intercalation of electrolyte ions. Such a phase transition enhances electronic conductivity and facilitates faster charge transfer.

Simultaneously, metal oxides, possessing intrinsic metallic conductivity and rich redox activity, provide substantial faradaic capacitance, thereby enhancing the overall energy storage capacity of the composite. Furthermore, the existence of several oxidation states facilitates subsequent redox reactions during cycling, thereby providing a broader potential window and further improving electrochemical performance.^142^ A PPy/MoS_2_/rGO ternary composite exhibited a remarkably high capacitance of 1561 F g^−1^ at 1 A g^−1^, highlighting the synergistic effect of hybrid components.^143^ Similarly, a MoS_2_@PANI hybrid delivered 645 F g^−1^ with 89% retention after 2000 cycles at 10 A g^−1^.^144^ Other ternary composites, such as MoS_2_/PANI/CNT and MoS_2_/MoO_2_/PAN, also show improved performance compared to their individual components. A PPy/MoS_2_/rGO ternary composite exhibited a remarkably high capacitance of 1561 F g^−1^ at 1 A g^−1^, highlighting the synergistic effect of hybrid components.^143^ Similarly, a MoS_2_@PANI hybrid delivered 645 F g^−1^ with 89% retention after 2000 cycles at 10 A g^−1^.^144^ Overall, this comparative overview underscores the potential of structural engineering, hetero-component integration, and phase control (1T *vs.* 2H) in tailoring MoS_2_-based materials for advanced supercapacitor technologies.

Table 4 presents a comparative analysis of MoS_2_-based electrode materials documented for supercapacitor applications, highlighting their morphology, synthesis approaches, and electrochemical performance. The table is structured to illustrate the impact of various structural modifications influence the charge-storage behaviour of MoS_2_. As summarised, pristine 2H-MoS_2_ generally shows limited electrochemical performance due to its semiconducting nature, low intrinsic electrical conductivity, and restricted interlayer spacing, which together hinder ion diffusion and redox activity. Conversely, metallic 1T-MoS_2_ exhibits markedly superior performance, with specific capacitances in the range of 650–710 F g^−1^ and cycling stability of up to 95% after 10 000 cycles. MoS_2_-based composites, particularly those integrated with metal oxides, conductive carbons, and conductive polymers, further improve electrochemical behaviour by increasing conductivity, exposing more active sites, and facilitating ion transport, resulting in specific capacitances typically higher than pristine 2H-MoS_2_. The comparison highlights that while 2H-MoS_2_ provide mechanical and chemical stability, its capacitive performance remains moderate (280–576 F g^−1^, stability >80%), and substantial enhancement requires phase engineering or hybrid/composite strategies. This analysis underscores the current performance disparity and indicates that additional investigation into controlled phase modulation, lattice engineering, and scalable composite fabrication methods is essential to comprehensively comprehend and enhance the function of MoS_2_ in high-performance supercapacitors.

**Table 4:** Summary of MoS_2_-based electrode materials for supercapacitors

Material topography	Composition/phase type	Synthesis method	Specific capacitance (F g^−1^)@current density	Cycling stability	References
MoS_2_ nanosheet	2H-MoS_2_	Hydrothermal	129.2@1 A g^−1^	85.1 after 500 cycles	145
2H-MoS_2_ nanosheets	2H-MoS_2_	Hydrothermal	142.3@1 A g^−1^	87.1 after 5000 cycles	135
3D MoS_2_ nanoflowers	2H-MoS_2_	Hydrothermal + annealing	280@0.8 A g^−1^	89% after 4000 cycles	112
Monolayer MoS_2_ nanosheets	1T-MoS_2_	Chemical exfoliation + phase transition	650@0.5 A g^−1^	95% after 10 000 cycles	85
Hollow MoS_2_ nanospheres	Mixed 1T/2H-MoS_2_	One-pot solvothermal	405@1 A g^−1^	92% after 3000 cycles	146
MoS_2_/rGO composite	Mixed 1T/2H-MoS_2_ + rGO	Hydrothermal + freeze drying	387.6@1.2 A g^−1^	90% after 5000 cycles	147
MoS_2_/CNT hybrid	2H-MoS_2_ + CNT	Hydrothermal	436@1 A g^−1^	96% after 1000 cycles	140
MoS_2_/NiO nanocomposite	2H-MoS_2_ + NiO	Hydrothermal	1048.75@1 A g^−1^	80% after 5000 cycles	148
MoS_2_@PANI hybrid	2H- MoS_2_ + PANI	Hydrothermal	645@0.5 A g^−1^	89% after 2000 cycles@10 A g^−1^	144
MoS_2_/MoO_2_/PAN composite	MoS_2_ + MoO_2_ + PAN	Solvothermal + heat treatment	439.8@1 A g^−1^	98.9% after 2000 cycles	149
MoS_2_/PANI/CNT composite	MoS_2_ + PANI + CNT	Polymerization	245 F cm^−3^@0.3 A cm^−3^	80% after 1000 cycles	150
PPy/MoS_2_/rGO composite	MoS_2_ + PPy + rGO	Hydrothermal	1561@1 A g; 786@15 A g^−1^	76% after 5000 cycles	143
NiCo_2_S_4_/MoS_2_ nanocomposites	NiCo_2_S_4_ + MoS_2_	Solvothermal	2594@0.8 A g^−1^	102% over 15 000 cycles	151

## 4 Conclusion

Among the wide range of materials explored for supercapacitor applications, two-dimensional transition metal dichalcogenides (2D TMDs), especially molybdenum disulfide (MoS_2_), have gotten a lot of attention because of their unique layered structure, large specific surface area, and tunable electronic, optical, and electrochemical properties. The electrochemical performance of MoS_2_ as a supercapacitor electrode is highly dependent on both its structural phase and layer number. While bulk MoS_2_ exhibits an indirect bandgap, the monolayer form transitions to a direct bandgap, resulting in enhanced charge transport and optoelectronic characteristics.

Different polymorphic phases of molybdenum disulfide exist, with unique atomic configurations and characteristics. The most prominent polymorphs are the semiconducting 2H phase, the metallic 1T phase, and the metastable 3R phase. Among these, 1T MoS_2_ has higher conductivity and is more favorable for the application of energy storage. Improved conductivity and charge storage capacities can be achieved through controlled phase transitions. These transitions can be done through elemental doping, conducting polymers, or hybridisation with redox-active materials. In summary, 2D MoS_2_ offers highly versatile properties for next-generation supercapacitors, owing to its customizable electrical characteristics, polymorphic diversity, and rich surface chemistry. However, significant issues persist, such as restricted electrical conductivity in some stages, structural instability during cycling, and obstacles in scalable, cost-effective synthesis. Future studies should emphasize the advancement of MoS_2_-based composites, the real-time observation of phase transitions, and the implementation of ecologically sustainable production methods. Overcoming these issues will expedite the development of high-performance, durable MoS_2_-based supercapacitors for sustainable energy storage applications.

## Conflicts of interest

The authors declare that there are no conflicts of interest.

## Data Availability

The data supporting this review are available from the articles cited within the manuscript. No new data were generated in this work.
